# Elucidating the mechanism of the Ley–Griffith (TPAP) alcohol oxidation†
†Electronic supplementary information (ESI) available. See DOI: 10.1039/c7sc04260d


**DOI:** 10.1039/c7sc04260d

**Published:** 2017-10-17

**Authors:** Timothy J. Zerk, Peter W. Moore, Joshua S. Harbort, Sharon Chow, Lindsay Byrne, George A. Koutsantonis, Jeffrey R. Harmer, Manuel Martínez, Craig M. Williams, Paul V. Bernhardt

**Affiliations:** a School of Chemistry and Molecular Biosciences , University of Queensland , Brisbane 4072 , Queensland , Australia . Email: p.bernhardt@uq.edu.au ; Email: c.williams3@uq.edu.au; b Centre for Advanced Imaging , University of Queensland , Brisbane 4072 , Australia; c Centre for Microscopy, Characterisation and Analysis , University of Western Australia , Crawley , Western Australia 6009 , Australia; d School of Molecular Sciences , University of Western Australia , Crawley , Western Australia 6009 , Australia; e Departament de Química Inorgànica I Orgànica , Secció de Química Inorgànica , Universitat de Barcelona , Martí i Franquès 1-11 , E-08028 Barcelona , Spain

## Abstract

The Ley–Griffith reaction is utilized extensively in the selective oxidation of alcohols to aldehydes or ketones. The central catalyst is commercially available tetra-*n*-propylammonium perruthenate (TPAP, *n*-Pr_4_N[RuO_4_]) which is used in combination with the co-oxidant *N*-methylmorpholine *N*-oxide (NMO). Although this reaction has been employed for more than 30 years, the mechanism remains unknown. Herein we report a comprehensive study of the oxidation of diphenylmethanol using the Ley–Griffith reagents to show that the rate determining step involves a single alcohol molecule, which is oxidised by a single perruthenate anion; NMO does not appear in rate law. A key finding of this study is that when pure *n*-Pr_4_N[RuO_4_] is employed in anhydrous solvent, alcohol oxidation initially proceeds very slowly. After this induction period, water produced by alcohol oxidation leads to partial formation of insoluble RuO_2_, which dramatically accelerates catalysis *via* a heterogeneous process. This is particularly relevant in a synthetic context where catalyst degradation is usually problematic. In this case a small amount of *n*-Pr_4_N[RuO_4_] must decompose to RuO_2_ to facilitate catalysis.

## Abstract 2

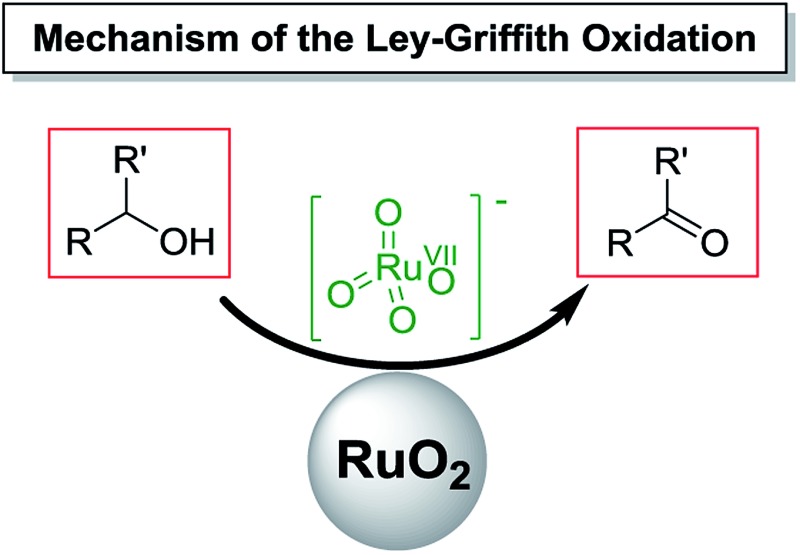
The mechanism of the Ley–Griffith alcohol oxidation has been elucidated using time-resolved spectroscopic methods.

## Introduction

The controlled oxidation of a primary alcohol to an aldehyde is a fundamentally important reaction deployed in academic and industrial settings^1^ to access versatile chemical building blocks, synthetic intermediates, and final targets. Amongst the multitude of reagents and conditions available to perform this functional group transformation,^2^,^3^ selectivity (*i.e.* avoiding over-oxidation) and versatility (*i.e.* tolerant of other functional groups) are key criteria.^4^

Historically, one-step alcohol oxidations to aldehydes, have relied heavily on chromium reagents (*e.g.* pyridinium chlorochromate^5^ (PCC)) activated sulfur protocols (*e.g.* Swern^6^,^7^ and Corey–Kim^8^) and manganese compounds (*e.g.* MnO_2_).^9^ More recently developed methods include hypervalent iodine compounds (*e.g.* Dess–Martin periodinane (DMP)^10^,^11^ followed by 2-iodoxybenzoic acid (IBX)^12^–^14^), and nitroxyl radicals (*e.g.* (2,2,6,6-tetramethyl-piperidin-1-yl)oxyl (TEMPO)^15^ followed by 2-azaadamantane *N*-oxyl (AZADO)^16^). In addition, molecular oxygen based methods using transition metals^17^–^20^ have also appeared in significant numbers. On balance, however, the mainstay protocols that dominate the one-step alcohol oxidation landscape are Swern, IBX/DMP, TEMPO and Ley–Griffith oxidations.^21^ The Ley–Griffith reaction followed on from early work by Sharpless reporting that ruthenium complexes (*e.g.* [Ru(PPh_3_)_3_Cl_2_]) catalyzed one-step oxidation of alcohols.^22^

**Scheme 1:**
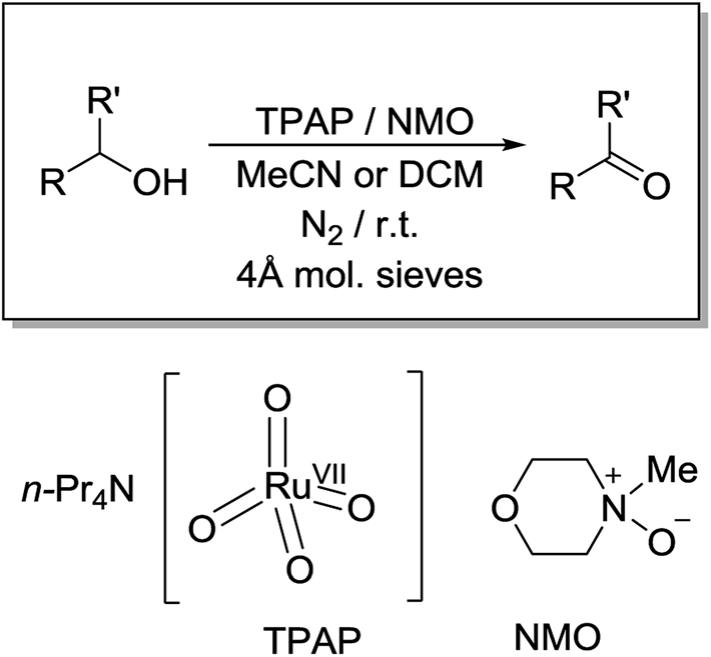
Reaction conditions of the Ley–Griffith alcohol oxidation.

The Ley–Griffith oxidation^21^,^23^–^28^ utilizes the catalyst *n*-Pr_4_N[RuO_4_] in combination with an excess (1.5 equivalents) of the co-oxidant *N*-methylmorpholine *N*-oxide (NMO – Scheme 1), both available commercially. Like many named reactions, modifications of the Ley–Griffith reaction have been reported^29^–^37^ (*e.g.* NMO·TPB^38^ and DABCOO·TPB^39^ from our own laboratory), as have numerous alternative applications using TPAP as a catalyst.^40^–^51^

Since the initial report of *n*-Pr_4_N[RuO_4_]-catalyzed alcohol oxidation some 30 years ago only Lee *et al.*^52^,^53^ have attempted to elucidate the mechanism. However, the work of Lee was focused on the sub-stoichiometric *n*-Pr_4_N[RuO_4_] alcohol oxidation (*i.e.* in the absence of the essential co-oxidant NMO) and was confined to a single spectroscopic method (UV-Vis). Under these conditions *n*-Pr_4_N[RuO_4_] decomposed to insoluble ruthenium dioxide which obscured UV-Vis absorption spectra of both the [RuO_4_]^–^ chromophore and the oxidized organic product. This work is quoted throughout the literature associated with the Ley–Griffith oxidation,^23^–^25^ stating that the mechanism is auto-catalytic and noting that the overall reaction appears to involve a three-electron reduction of *n*-Pr_4_N[RuO_4_].^24^

Perruthenate exhibits a diversity of reactivity depending primarily on solvation. In aqueous solution it has been proposed to react *via* a single electron process,^54^ based on its stoichiometric oxidation of cyclobutanol, which led to ring-opened products. This was evidence of radical formation at the α-carbon, and a corresponding low yield (33%) of cyclobutanone was found. When cyclobutanol was oxidized with *n*-Pr_4_N[RuO_4_]/NMO in dichloromethane Ley and Griffith observed cyclobutanone as the product in a 95% yield, indicating that a clean 2-electron oxidation is mediated by perruthenate under these conditions.^21^

We recently demonstrated that the role of NMO is to rescue the highly reactive Ru^V^ form of the catalyst by rapidly reoxidising it to [RuO_4_]^–^, thus sustaining catalysis.^55^ Others have suggested that NMO is required to access the true catalyst, in forming a perruthenate–NMO adduct.^56^ Clearly, the catalyst, co-oxidant and the substrate (*i.e. n*-Pr_4_N[RuO_4_], NMO and alcohol) are all essential yet the function of each in the oxidation reaction (producing aldehyde/ketone) has not previously been investigated. In view of the popularity that the Ley–Griffith reaction commands in synthesis, and the lack of mechanistic understanding, we initiated an extensive spectroscopic evaluation, which has resolved important mechanistic details regarding the catalytic cycle of this key reaction.

## Experimental

Reagents, equipment and synthetic details are given in the ESI.†

### UV-Vis monitored reaction kinetics

In a typical experiment, 2.0 mL of a fresh solution of *n*-Pr_4_N[RuO_4_] (0.25 mM) was added to a 1 cm UV-Vis quartz cuvette and thermostatted at 303 K. After 10 minutes, NMO was added from a concentrated stock solution (0.85 M in MeCN) pre-dried over molecular sieves for 16 h. Finally, diphenylmethanol was added from a stock solution (0.1 M in MeCN) to initiate the reaction. The maximum reaction rate (*v*_max_) was obtained in each case from a tangent to the steepest portion of the time-resolved single wavelength profile at 336 nm (ESI Fig. S2†). The slopes of the log–log plots of *v*_max_ against the initial concentrations of *n*-Pr_4_N[RuO_4_], NMO and diphenylmethanol revealed the reaction order of each reagent. For alcohol-dependent kinetic measurements the reference cuvette contained a solution of 0.25 mM *n*-Pr_4_N[RuO_4_] in MeCN such that the constant spectrum of the catalyst was subtracted. This allowed a greater range of alcohol concentrations to be exploited without flooding the spectrophotometer detector. Nevertheless the detector was still saturated by absorbance from the benzophenone product for experiments utilizing high initial concentrations of diphenylmethanol, but this did not affect the accurate determination of *v*_max_.

## Results

### Time-resolved UV-Vis spectroscopy

**Fig. 1:**
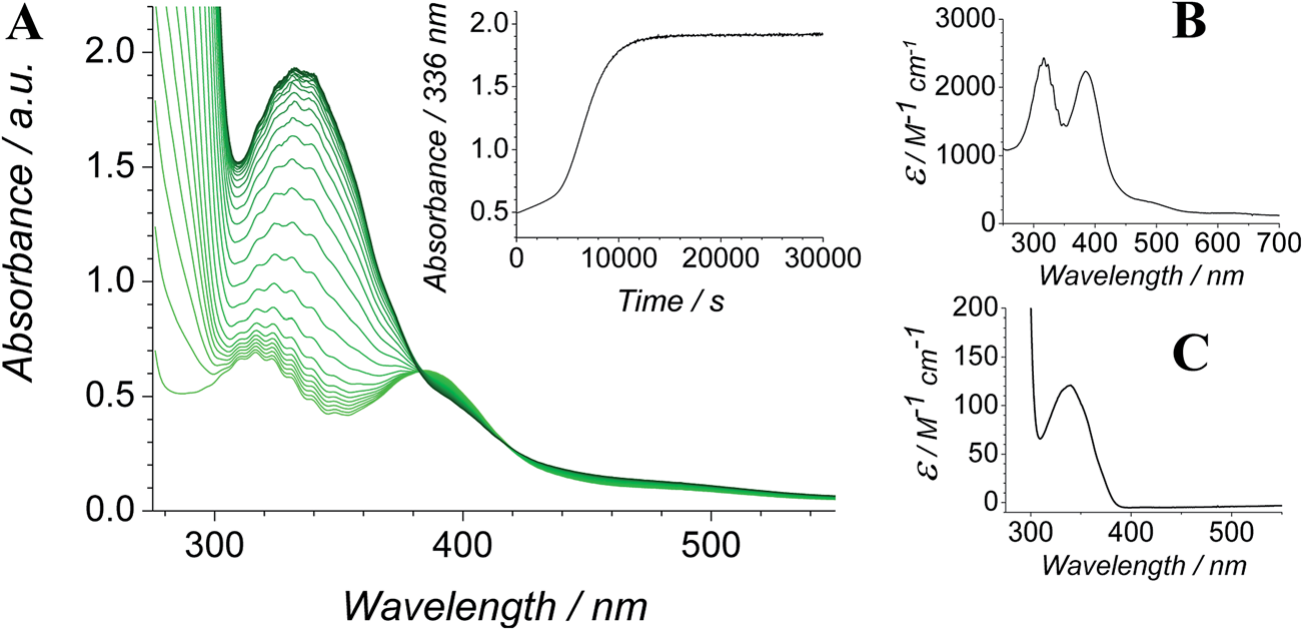
(A) Time-resolved UV-Vis spectra following the oxidation of 12.5 mM diphenylmethanol by 0.25 mM *n*-Pr_4_N[RuO_4_] and 67 mM NMO in MeCN (303 K). Spectra are displayed at ten minute intervals over the course of 8 h. Inset – absorbance profile at 336 nm. (B) UV-Vis spectrum of *n*-Pr_4_N[RuO_4_] in MeCN. *λ*_max_ 316 nm (*ε* 2430 M^–1^ cm^–1^) and 385 nm (*ε* 2230). (C) UV-Vis spectrum of benzophenone in MeCN. *λ*_max_ 336 nm (*ε* 120 M^–1^ cm^–1^).

The Ley–Griffith alcohol oxidation reaction was followed using time-resolved UV-Vis spectroscopy (Fig. 1). A large excess of NMO co-oxidant (pre-dried over 4 Å molecular sieves) was added to ensure the regeneration of perruthenate during catalysis. We have recently shown how NMO rescues the unstable reduced Ru^V^ catalyst by reoxidation to [RuO_4_]^–^.^55^ Diphenylmethanol was selected as the alcohol substrate because it does not absorb above 280 nm while the corresponding ketone (benzophenone) absorbs at 336 nm (*ε* 120 M^–1^ cm^–1^ – Fig. 1C) and at 280 nm (*ε* 2910).^56^ This allows a quantitative and straightforward analysis of product formation *in situ* without the need for chromatography or mass spectrometry. Additionally, diphenylmethanol is a secondary alcohol so over-oxidation to a carboxylic acid is avoided.

Typical alcohol oxidation experiments proceeded to completion over the course of several hours (Fig. 1A). Although the emerging benzophenone chromophore dominates the profile of Fig. 1A, the distinctive spectral features of the perruthenate anion at 316 nm and 385 nm remain throughout the reaction. This confirms that perruthenate is a catalytic, not stoichiometric, reagent and is at steady state throughout. Subtracting the ‘baseline’ spectrum of perruthenate (Fig. 1B) leads to ESI Fig. S3† which, along with the extinction coefficient of benzophenone at 336 nm, reveals that the alcohol is quantitatively converted to the ketone (Fig. S3† – inset).

**Fig. 2:**
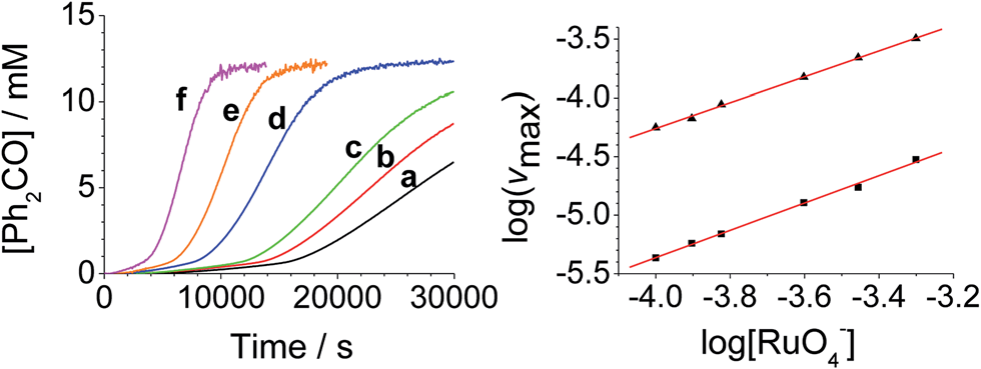
[RuO_4_]^–^-dependent kinetics (Left). Concentration profile for benzophenone during a reaction between *n*-Pr_4_N[RuO_4_], 150 mM NMO and 12 mM diphenylmethanol in MeCN (*T* = 303 K) (a) black – 0.100 mM, (b) red – 0.125 mM, (c) green – 0.150 mM, (d) blue – 0.250 mM, (e) orange – 0.350 mM, (f) pink – 0.500 mM *n*-Pr_4_N[RuO_4_]. (Right) log–log plots for the first (■ – slope = 1.1) and second (▲ – slope = 1.0) phases.

**Fig. 3:**
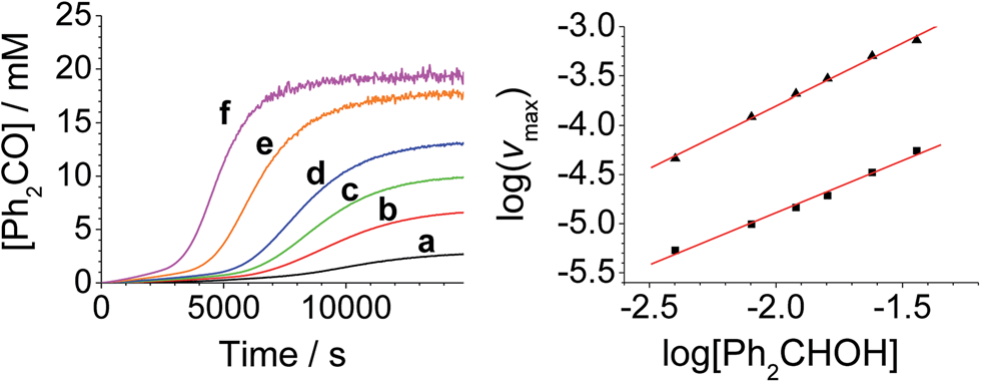
[Diphenylmethanol]-dependent kinetics (Left) concentration profile for benzophenone during a reaction between 0.25 mM *n*-Pr_4_N[RuO_4_], 150 mM NMO and diphenylmethanol in MeCN (*T* = 303 K). (a) Black – 4.0 mM, (b) red – 8.0 mM, (c) green – 12.0 mM, (d) blue – 16.0 mM, (e) orange – 24.0 mM, (f) pink – 36 mM diphenylmethanol. (Right) log–log plots for the first (■ – slope = 1.1) and second (▲ – slope = 1.2) phases.

**Fig. 4:**
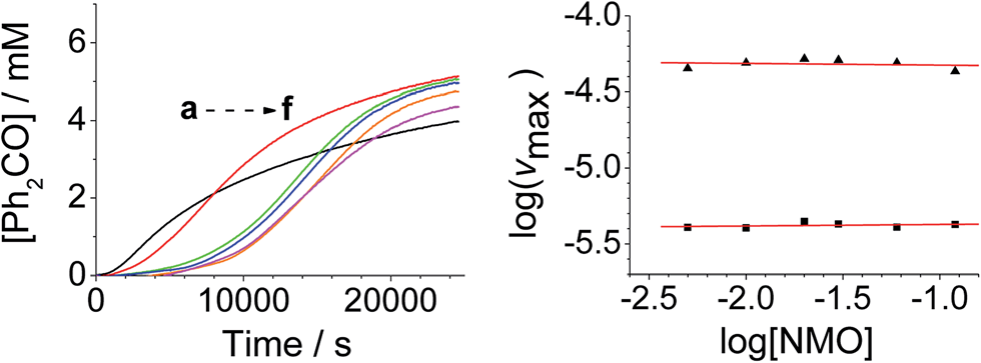
[NMO]-dependent kinetics (Left) concentration profile for benzophenone during a reaction between 0.25 mM *n*-Pr_4_N[RuO4], 6.0 mM diphenylmethanol and NMO in MeCN (*T* = 303 K). (a) Black – 5.0 mM, (b) red – 10.0 mM, (c) green – 20.0 mM, (d) blue – 30.0 mM, (e) orange – 60.0 mM, (f) pink – 120 mM NMO. Note: the different behavior with 5 mM NMO is due to an excess of alcohol *versus* NMO. (Right) log–log plots for the first (■) and second (▲) phases.

The concentration *versus* time profile for the oxidation (inset of Fig. 1A) is unusual in showing two distinct linear phases before the reaction is complete at ∼10 000 s (∼2¾ h). The reaction initially proceeds slowly through the first hour where an induction phase is apparent (0–4000 s), and only a small proportion (∼10%) of the product is formed. At ∼5000 s the reaction rate undergoes a ten-fold acceleration and the reaction proceeds rapidly and quantitatively to completion. No satisfactory kinetic model could reproduce the time-resolved absorbance changes. However, the overall reaction could be separated into its slow and fast catalytic phases, which were each analyzed independently in terms of their reagent concentration dependence. The steady state catalytic rate of alcohol oxidation (*v*_max_) was obtained from the slope of the linear portions of the slow and rapid phases (see Fig. S2†). The reaction order with respect to perruthenate, alcohol and NMO was determined by plotting the logarithm of the reaction velocity (during the linear regions of the slow and fast phase) *versus* the logarithm of reagent concentration. The resulting concentration dependent profiles and log–log plots are shown in Fig. 2–4.

From the data it is apparent that the rate law for both phases of the reaction is identical; first order in perruthenate, first order in alcohol and zero order with respect to NMO (eqn (1a) and (1b)). It has been shown previously that perruthenate is an effective oxidant on its own,^21^,^54^,^57^ which suggests that NMO is not required to access the true catalyst. The zero-order dependence of *v*_max_ on the concentration of NMO confirms this.

1a *v*_max_ = *k*[RuO_4_^–^][ROH] – (induction phase)

1b *v*′_max_ = *k*′[RuO_4_^–^][ROH] – (rapid phase)

These findings are not consistent with the work of Lee *et al.* who reported the rate law as second order with respect to perruthenate based on its decomposition to RuO_2_.^53^ Nevertheless, our reaction mechanism does not explain why there are two distinct phases to the reaction *i.e.* there are two distinct values for the bimolecular rate constants in eqn (1a) and (1b). The observed behavior suggests that, as the oxidation proceeds, one of the products accelerates the reaction *i.e.* the reaction is autocatalytic. Indeed when higher initial concentrations of alcohol and perruthenate are employed, an initially faster rate of oxidation leads to a surge of product formation, the induction period being much shorter.

**Fig. 5:**
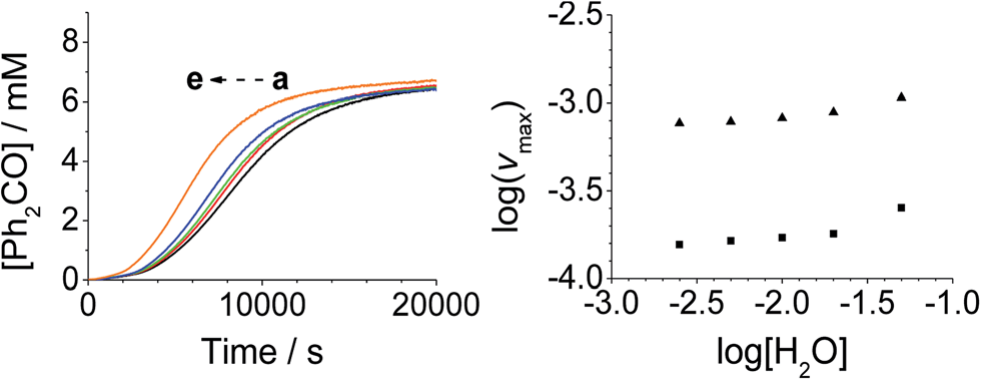
[H_2_O]_0_-dependent kinetics (Left) concentration profile for benzophenone during a reaction between 0.25 mM *n*-Pr_4_N[RuO_4_], 6.0 mM diphenylmethanol and 20 mM NMO in MeCN (*T* = 303 K). (a) Black – 2.5 mM, (b) red – 5.0 mM, (c) green –10.0 mM, (d) blue – 20.0 mM, (e) orange – 50.0 mM H_2_O added at *t*_0_. (Right) log–log plots for the first (■) and second (▲) phases.

Addition of either benzophenone or *N*-methyl morpholine at the start of the reaction had no effect on the kinetics; the concentration–time profiles were identical to those shown in Fig. 1–4. The effect of the addition of water was also studied (being a significant factor given the fact that the Ley–Griffith oxidation requires molecular sieves to drive the reaction to completion) by adding different concentrations at the start of the reaction (Fig. 5). The near-zero slope of the log–log plots up to 10 mM H_2_O indicates that water is also not involved in the rate law. However, when much higher water concentrations were added (>40 mM), the induction period was truncated and the maximum rate was also slightly increased.

### EPR spectroscopy

**Fig. 6:**
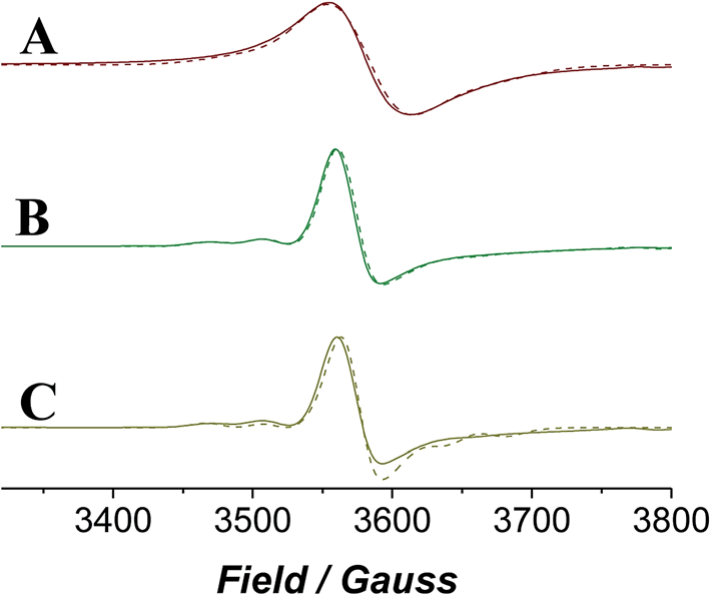
X-band (*ν*_av_ = 9.696 GHz) CW EPR spectra measured for *n*-Pr_4_N[RuO_4_] in acetonitrile, with differing additives. (A) Neat *n*-Pr_4_N[RuO_4_], (B) *n*-Pr_4_N[RuO_4_] + NMO, (C) *n*-Pr_4_N[RuO_4_] + NMO + diphenylmethanol. *T* = 6 K for all. Experimental spectra are solid lines and simulated spectra are the broken lines.

EPR spectroscopy provides a lens through which the paramagnetic catalyst alone is viewed as the organic components do not contribute to the spectra. The continuous wave (CW) EPR spectrum of a frozen solution of *n*-Pr_4_N[RuO_4_] in MeCN was measured at X-band frequency (Fig. 6) and simulated with EPR50F^58^ to reveal a slightly distorted d^1^ tetrahedral complex (*g*_*x*,*y*_ = 1.937, *g*_*z*_ = 1.910). These *g*-values (Table 1) are comparable with those measured for other distorted d^1^ tetraoxidoanions: [CrO_4_]^3–^*g*_*x*_ = 1.84, *g*_*y*_ = 1.85, *g*_*z*_ = 1.94;^59^ [MnO_4_]^2–^*g*_*x*_ = 1.98, *g*_*y*_ = 1.97, *g*_*z*_ = 1.94;^60^ [ReO_4_]^2–^*g*_*x*,*y*_ = 1.72, *g*_*z*_ = 1.85.^61^

**Table 1:** Experimental spin Hamiltonian parameters for *n*-Pr_4_N[RuO_4_] in acetonitrile, and after addition of NMO and diphenylmethanol

Sample	*g*_*x*_	*g*_*y*_	*g*_*z*_	*A*_*x*_	*A*_*y*_	*A*_*z*_ ^*a*^ (G)
*n*-Pr_4_N[RuO_4_]	1.937	1.937	1.910	∼90	∼90	—
*n*-Pr_4_N[RuO_4_] + NMO	1.938	1.938	1.910	∼110	∼110	—
*n*-Pr_4_N[RuO_4_] + NMO + Ph_2_CHOH	1.939	1.936	1.918	∼110	∼110	—

^*a*^ ^101^Ru, *I* = 5/2, abundance 17.06%; ^99^Ru, *I* = 5/2, abundance 12.76%; — indicates the value is undetermined.

When NMO was added to *n*-Pr_4_N[RuO_4_] the EPR signal became slightly more anisotropic and peaks from hyperfine coupling to ^99^Ru (13%) and ^101^Ru (17%) (both *I* = 5/2) were resolved (Fig. 6). However, the spectrum lacked any discernible superhyperfine coupling to the ^14^N (*I* = 1) nucleus of NMO. Inner sphere coordination of NMO can be discounted as this would change the geometry and symmetry of the complex and would be accompanied by a more significant effect on the spin Hamiltonian parameters than is observed in Fig. 6. The reluctance of perruthenate to expand its coordination number above four is not unexpected for a tetrahedral oxidoanion and is demonstrated by its persistent optical spectrum in a range of solvents.^52^,^55^,^62^ When NMO was added to [RuO_4_]^–^ the UV-Vis spectrum was similarly unaffected (Fig. S4†). The subtle changes observed in the EPR spectrum must arise from weak outer-sphere interactions. When diphenylmethanol was added to the mixture of [RuO_4_]^–^ and NMO (*i.e.* under Ley–Griffith conditions) then rapidly frozen the CW EPR spectrum did not change either (Fig. 6C); there is no evidence of an alcohol complex of Ru^VII^.

**Fig. 7:**
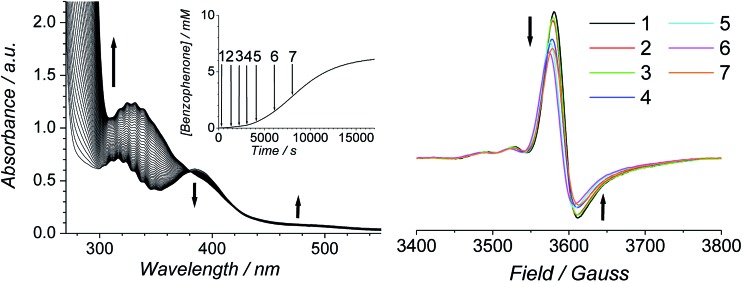
(Left) UV-Vis and concentration profile for benzophenone (inset) during a reaction between 0.25 mM *n*-Pr_4_N[RuO_4_], 6.0 mM diphenylmethanol and 60 mM NMO in MeCN (*T* = 303 K). (Right) Frozen X-band spectra (*ν*_av_ = 9.7041 GHz) measured at the intervals indicated in the inset (*T* = 6 K).

The progress of the Ley–Griffith oxidation was followed by parallel EPR/UV-Vis spectroscopy. At various intervals, a small amount of the reaction mixture in the UV-Vis cell was removed, frozen and the EPR spectrum measured. A lower concentration of alcohol was chosen to slow the reaction so that several measurements could be taken during both the induction and rapid phases (Fig. 7). Throughout the induction period, the only notable change to the EPR was a small decrease in perruthenate signal intensity; furthermore, when the oxidation reached its maximum rate (*ca.* 8000 s), ∼25% of the perruthenate signal had been lost. No new EPR peaks appeared during this time and the field positions of the existing peaks did not change. Given the fact that both Ru^VI^ (d^2^) and Ru^V^ (d^3^) are EPR-active and adopt different coordination geometries from perruthenate^63^,^64^ the results in Fig. 7 indicate the absence of both species. As a whole, these observations indicate that perruthenate is partially converted into an EPR-silent species with a featureless UV-Vis spectrum during the induction period.

In the absence of NMO, perruthenate is irreversibly reduced by alcohols or water and insoluble ruthenium dioxide dihydrate (RuO_2_·2H_2_O) is the product.^52^,^53^,^57^,^62^,^65^,^66^ This is observed as a fine black precipitate which produces a featureless, baseline-shifted UV-Vis spectrum; no EPR spectrum of this species has been reported. Indeed when diphenylmethanol was reacted with *n*-Pr_4_N[RuO_4_] in a 1 : 1 stoichiometric ratio in the absence of NMO the same outcome was achieved. The reaction was followed by EPR spectroscopy and samples of the mixture were taken every minute then frozen before their spectrum was measured. After 2 minutes the initially yellow-green perruthenate solution had become a black suspension and the sample was EPR silent (ESI Fig. S5†).

Therefore, despite the high initial concentration of NMO, an appreciable amount of RuO_2_·2H_2_O forms during the induction phase in order to account for the ∼25% decrease in the perruthenate EPR signal (Fig. 7 (right)). The formation of RuO_2_·2H_2_O also explains the slight baseline shift observed in the optical spectrum during the reaction and the small decrease in the perruthenate peak at 385 nm (see arrows in Fig. 7 (left) as well as Fig. 1A).

### ^99^Ru NMR

We also anticipated that ^99^Ru NMR could be useful for following the catalyst throughout the oxidation. It was expected that the relatively symmetrical nature of the [RuO_4_]^–^ anion would provide the opportunity to observe a signal in spite of the low receptivity and quadrupole moment. Unfortunately across an exhaustively searched sweep width we were unable to obtain a discernible ^99^Ru NMR signal from *n*-Pr_4_N[RuO_4_].

### RuO_2_·2H_2_O is a co-catalyst

**Fig. 8:**
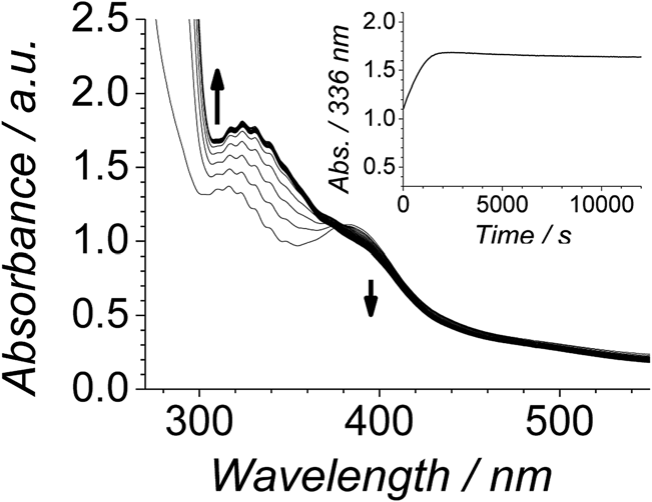
Time-resolved spectra following the oxidation of 6.0 mM diphenylmethanol by 0.25 mM *n*-Pr_4_N[RuO_4_] and 60 mM NMO in MeCN (*T* = 303 K) with 16 μL of RuO_2_·2H_2_O stock solution added at *t*_0_. Spectra are displayed at five minute intervals. Inset – single wavelength profile at 336 nm.

The effect of RuO_2_·2H_2_O was consequently explored by comparing parallel Ley–Griffith oxidations of diphenylmethanol with and without added ruthenium dioxide (see ESI† for preparative details). The results are shown in Fig. 8. When RuO_2_·2H_2_O was added at the start of the reaction (inset) the induction period was bypassed and the oxidation proceeded rapidly and smoothly to completion. The contribution of this additive to the absorption spectrum can be seen by comparing Fig. 1 and 7 (no RuO_2_·2H_2_O) with Fig. 8 (added RuO_2_·2H_2_O). However, the net change in absorbance at 336 nm (due to benzophenone formation) is equivalent in both cases. Overall, these results indicated that addition of RuO_2_·2H_2_O bypasses the slow induction phase, but the yield of product remains the same.

### ^18^O-enriched alcohol

The possibility of O-atom transfer from perruthenate to the carbonyl product was studied using ^18^O-labelled piperonol (78% enriched) as a substrate and naturally abundant (^16^O) perruthenate and NMO (see ESI†). After standard Ley–Griffith oxidation ^18^O enrichment in the product piperonal was 68% which is not significantly different from the starting material (Fig. S6†). Mass spectroscopy of an aliquot of the crude reaction solution after the oxidation also showed the presence of naturally abundant NMO and [RuO_4_]^–^ with no incorporation of ^18^O. These observations rule out any possibility of O-atom transfer from perruthenate to alcohol.

## Discussion

Notwithstanding the complicated kinetic profile of the Ley–Griffith oxidation (Fig. 1A) its rate law has been established (eqn (1a) and (1b)) and only involves perruthenate and alcohol. Furthermore, the same reaction order persists during both the slow initial phase and the faster second phase. Although ruthenium dioxide accelerates the oxidation, it is not an effective oxidant. Indeed a comprehensive study by Nobuko and Masakatsu has already demonstrated that RuO_2_ and RuO_2_·2H_2_O are ineffective oxidants of unactivated alcohols.^67^

**Scheme 2:**
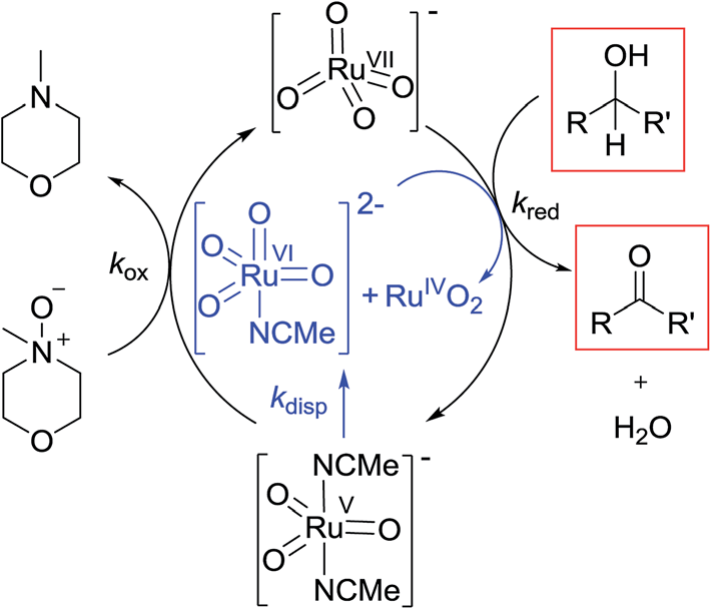
Mechanism proposed for the Ley–Griffith alcohol oxidation in acetonitrile.

Ruthenium dioxide is indirectly formed by the concerted, two-electron reduction of perruthenate (*k*_red_) to the highly unstable Ru^V^ which disproportionates (*k*_disp_) to RuO_2_·2H_2_O and ruthenate(vi) (Scheme 2). Ruthenate(vi) is also capable of alcohol oxidation and so may undergo a second reaction with alcohol to generate more RuO_2_·2H_2_O.^54^,^68^ Therefore, any Ru^V^ that undergoes disproportionation in the presence of alcohol is quantitatively converted to RuO_2_·2H_2_O.2
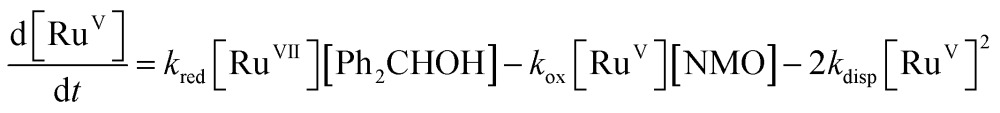



Evidently ruthenium dioxide provides an active surface upon which oxidation takes place *i.e.* catalysis moves from a purely homogeneous to a heterogeneous regime. Given the fact that both perruthenate and alcohol appear in the rate law during the accelerated second phase (eqn (1b)), both species must be adsorbed onto the surface of the suspended RuO_2_·2H_2_O (*i.e.* a Langmuir-type mechanism). This is analogous to the oxidations of alcohols and hydrocarbons by permanganate which are catalyzed by colloidal MnO_2_.^69^,^70^

Under Ley–Griffith oxidation conditions, excess NMO limits Ru^V^ disproportionation by competitively re-oxidizing Ru^V^ to perruthenate (rate constant *k*_ox_ in eqn (2)).^55^ However, as the reaction proceeds, more water is produced and the NMO concentration decreases. Both factors make Ru^V^ disproportionation (*k*_disp_ in eqn (2)) more competitive with Ru^V^ re-oxidation by NMO (Scheme 2). It is ultimately the balance of these two reactions which determines how quickly the rapid phase of the reaction is accessed. Furthermore, higher alcohol and perruthenate concentrations accelerate the (bimolecular) reaction (*k*_red_ in eqn (2)) and thus leading to a surge in Ru^V^ concentration which, at steady state, is compensated by more rapid disproportionation (last term of eqn (2)) and production of the RuO_2_·H_2_O co-catalyst. Accordingly, the rapid catalytic phase occurs sooner at higher concentrations of alcohol and *n*-Pr_4_N[RuO_4_] (Fig. 2 and 3), which is consistent with synthetic scale Ley–Griffith oxidation reactions. Conversely, higher concentrations of NMO prolong the induction period by keeping [Ru^V^] low (Fig. 4).

There are innumerable reports detailing the synthetic application of the Ley–Griffith protocol, but throughout the literature there is a curious lack of any reference to an induction period like that observed here (Fig. 1–5). Synthetic-scale oxidations typically utilize much higher concentrations of reagents ([ROH], [RuO_4_]^–^ ∼ 0.5 M, a 100-fold concentration increase)^25^ than those examined here which may explain the disparity; higher concentrations of alcohol and perruthenate being coupled to a short induction period.

However, there is an additional relevant explanation. The overwhelming majority of synthetic studies use commercially available (97% pure) *n*-Pr_4_N[RuO_4_]. When the oxidation of diphenylmethanol was repeated in our study using this commercial catalyst, instead of the pure synthesized product (Fig. S7†), no induction period was observed and the oxidation proceeded smoothly to completion. The baseline shift of the spectrum at *t*_0_ indicates that a small amount of insoluble material (ruthenium dioxide) is present in the commercially obtained catalyst (Fig. S8†). It is well known that *n*-Pr_4_N[RuO_4_] is sensitive to moisture and light and must be kept under argon (preferably in a refrigerator). Regardless of these precautions, *n*-Pr_4_N[RuO_4_] eventually degrades to a black solid with no catalytic activity. Even fresh bottles of commercial 97% pure *n*-Pr_4_N[RuO_4_] contain ruthenium dioxide and this is evidently most of the 3% impurity that is present (Fig. S8†). Interestingly, in practical terms, an induction period is undesirable, and by serendipitous fortune the commercial catalyst containing a small (∼3%) amount of impurity bypasses this complication. However, *n*-Pr_4_N[RuO_4_] is still the essential oxidant and complete degradation to inert RuO_2_ inactivates the catalyst.

## Conclusions

A mechanistic study of the oxidation of diphenylmethanol using the Ley–Griffith reagent reveals that the rate determining step involves a single alcohol molecule which is oxidized in a concerted two electron process by a single [RuO_4_]^–^ anion. NMO is not required to form the active oxidant, its role being stoichiometric as the oxidant which reactivates the Ru^V^ form of the catalyst to Ru^VII^. A major finding of this study is that ruthenium dioxide, formed during the reaction, or present initially through degradation of the *n*-Pr_4_N[RuO_4_] catalyst, acts as a heterogeneous co-catalyst for the reaction. When freshly crystallized *n*-Pr_4_N[RuO_4_] is utilized, the oxidation proceeds slowly at the beginning of the reaction until a sufficient concentration of solid ruthenium dioxide is formed by disproportionation of the Ru^V^ form of the catalyst. Ironically, slightly impure catalyst performs more predictably, without an induction phase, than the pure catalyst. Nevertheless, the overall yield of product under both conditions is the same. Disproportionation of Ru^V^ is accelerated by the presence of water (produced by the reaction), or high catalyst/alcohol concentrations.

## Conflicts of interest

The authors declare no conflicts of interest.

## Supplementary Material

Supplementary information
