# Supplementary material for: Elucidating the mechanism of the Ley–Griffith (TPAP) alcohol oxidation
Source: Chem Sci. 2017 Oct 17;8(12):8435–42. doi: 10.1039/c7sc04260d (PMC5863698; doi:10.1039/c7sc04260d)
Supplement: Supplementary file 1 [file SC-008-C7SC04260D-s001.pdf]

Electronic Supplementary Material (ESI) for Chemical Science.
This journal is © The Royal Society of Chemistry 2017

# Electronic Supporting Information

# Elucidating the Mechanism of the Ley-Griffith (TPAP) Alcohol Oxidation

Timothy J. Zerk,[a] Peter W. Moore,[a] Joshua S. Harbort,[b] Sharon Chow,[a] Lindsay Byrne,[c] George A. Koutsantonis,[d] Jeffrey R. Harmer,[b] Manuel Martínez,[e] Craig M. Williams[a,*] and Paul V. Bernhardt[a,*]

[a] School of Chemistry and Molecular Biosciences, University of Queensland, Brisbane 4072, Queensland, Australia

[b] Centre for Advanced Imaging, University of Queensland, Brisbane 4072, Australia

[c] Centre for Microscopy, Characterisation and Analysis, University of Western Australia, Crawley, Western Australia 6009, Australia

[d] School of Molecular Sciences, University of Western Australia, Crawley, Western Australia 6009, Australia

[e] Departament de Química Inorgànica I Orgànica, Secció de Química Inorgànica, Universitat de Barcelona, Martí i Franquès 1-11, E-08028 Barcelona, Spain

## Experimental Details

### Reagents

All reagents and solvents were purified prior to use according to literature methods.[1] Ruthenium(III) trichloride hydrate was obtained from Precious Metals Online. Tetra-*n*-propylammonium perruthenate was synthesised (see below) except for comparative experiments, which utilised a commercial sample, obtained from Sigma Aldrich (97% purity). Commercial benzophenone was recrystallised from ethyl acetate, affording a colourless crystalline solid. N-Methylmorpholine N-oxide monohydrate ($NMO{\cdot}H_2O$) was obtained by concentrating under reduced pressure a 50 wt/wt% aqueous solution of NMO (obtained from Sigma Aldrich). Anhydrous NMO solutions in $CD_3CN$ were obtained by drying a monohydrate hydrate solution over 4 Å molecular sieves for 24 hours. $^{18}O$-enriched water was obtained from Novachem.

### Physical Methods

Time-resolved UV-Vis spectra were acquired with an Agilent 8453 diode array spectrophotometer equipped with a multi-cell holder. The cell holder was maintained at 303 K throughout with a circulating thermostat. X-band CW EPR experiments were carried out on a Bruker XE580 spectrometer equipped with a cryogen-free variable temperature cryostat from Cryogenic limited (model PT415). The field was calibrated with DPPH (2,2-diphenyl-1picrylhydrazyl, g = 2.0036). A 1 kW amplifier was used. Spectra were recorded at 3 - 6 K. A 2-step phase cycle was employed. All manipulations of solutions for EPR spectroscopy were carried out under a nitrogen atmosphere within a Belle Technology anaerobic glovebox ($O_2$ < 20 ppm) using carefully dried reagents and solvents. IR spectra were measured on a Perkin Elmer FT-IR spectrometer (Spectrum 2000). Melting points were recorded and uncorrected measurements repeated three times using a Digimelt MPA161 SRS apparatus. GC-MS was recorded using a Shimadzu GCMS-QP5000 machine using a Restek Rtx® -5MS column and analysed using GCMS solutions v1.20. Microanalyses were performed

by the University of Queensland Microanalytical Service. $^{99}$Ru NMR spectra, in $CD_3CN$, were acquired on a Bruker AVANCE IIIHD spectrometer operating at 23.08 MHz using the 10mm PABBO Broadband probe. The spectra were referenced to external $K_4[Ru(CN)_6]$.

**Synthesis**

*n-$Pr_4N[RuO_4]$ (TPAP)*

A modified version of the original synthesis reported by Griffith *et al* was used.[2] Two three-necked round bottomed flasks were connected through a short, tubular glass bridge (Figure S1). The first flask was charged with tetra-*n*-propylammonium hydroxide solution (1.0 M, 1.25 mL), deionized water (2.5 mL) and sodium hydroxide solution (1.0 M, 10.0 mL). The second flask contained sodium periodate (375 mg, 1.43 mmol) dissolved in deionized water (10.0 mL) to which was added a ruthenium trichloride solution (374 mg in 2.5 mL of deionized water). The reaction flasks were stoppered, and the reaction stirred at room temperature for 16 h. During the reaction volatile $RuO_4$, which formed in the second flask, diffused into the tetra-*n*-propylammonium hydroxide solution and precipitated as a dark green solid of *n*-$Pr_4N[RuO_4]$, which was filtered off, washed with water (5 mL) and dried under vacuum (147 mg, 33%).

M.p. (Dec): 165.2 ◦C ; HRMS: $^{m}/_{z}$ for $^{102}RuO_4^-$, calcd: 165.8846, found: 165.8849; HRMS: $^{m}/_{z}$ for $C_{12}H_{28}N^+$, calcd: 186.2216, found: 186.2213; UV-Vis: $\lambda_{max}$ (MeCN) = 316, 385 nm; IR: $\nu_{max}$ 2969, 2940, 2879, 1476, 1457, 1390, 1039, 982, 968, 823, 750 $cm^{-1}$; Anal. Calcd for $C_{12}H_{28}NO_4Ru$: C, 41.01; H, 8.03; N, 3.99. Found: C, 41.12; H, 8.01; N, 3.98.

*Diphenylmethanol*

Benzophenone (1.999 g, 11.0 mmol) was dissolved in methanol (25 mL) and cooled to 0 °C (in an ice water bath). Sodium borohydride (951 mg, 22.0 mmol) was added and the reaction stirred

at 0 °C (in an ice water bath) for five minutes before being allowed to warm to room temperature and stirred for 20 minutes. The reaction was cooled to 0 °C (in an ice water bath) and diluted with water (25 mL), the reaction concentrated to half volume, then extracted with diethyl ether (3 × 30 mL). The organic fractions were combined, washed with brine, dried over sodium sulfate, passed through a silica plug (5 x 2 cm) and concentrated providing diphenylmethanol in 97% yield (1.970 g).

*$^{18}$O-labelled Piperonol*

Water-$^{18}$O (250 µL) was added to sodium hydroxide (96 mg, 2.4 mmol) in an oven-dried Schlenk flask that was evacuated and back flushed with argon. The mixture was stirred until all the hydroxide had dissolved. An ice-cold water bath was used to cool the flask during this process. Piperonyl chloride (61 mg, 0.36 mmol) in anhydrous THF (70 µL) was added in one-portion to the base. The reaction was stirred at 80 $^{o}$C for 16 h, after which time no starting material could be detected by GCMS. After extraction with anhydrous THF (3 times), the combined THF solutions were dried over anhydrous sodium sulfate and concentrated under reduced pressure. The desired product (10 mg, 19%) and dipiperonyl ether (26 mg, 26%) were isolated by flash chromatography (silica gel, 20% ethyl acetate in pet. spirit). $^{1}$H NMR data matched the literature.[3]

$^{1}$H NMR (500 MHz, $CDCl_3$) $\delta_H$ 6.89 - 6.86 (m, 1H), 6.83 - 6.80 (m, 1H), 6.78 (d, *J* = 7.8 Hz, 1H), 5.95 (s, 2H), 4.58 (d, *J* = 2.8 Hz, 2H); $^{13}$C NMR (100 MHz, $CDCl_3$) $\delta_C$ 147.8, 147.1, 134.9, 120.5, 108.2, 107.9, 101.0, 65.3; EIMS m/z (%): 51 (18), 62 (10), 63 (24), 65 (75), 66 (27), 77 (32), 93 (86), 94 (15), 96 (25), 121 (13), 122 (13), 123 (60), 124 (37), 135 (68), 151 (13), 152 (29), 153 (33), 154 (100). The ratio of $^{18}$O-piperonol to $^{16}$O-piperonol was determined from the intensities of the 154 and 152 m/z peaks (78%:22% $^{18}$O-piperonol:$^{16}$O-piperonol). The $^{16}$O-piperonol arose from naturally abundant $Na^{16}OH$ used in the synthesis. Theoretically the $^{18}$O:$^{16}$O ratio should be 83:17%.

*Piperonal (unlabelled and $^{18}$O-labelled)*

*N*-methylmorpholine *N*-oxide monohydrate (10.5 mg, 77.7 µmol), pre-dried under high vacuum for 1 h, was added to either unlabelled or $^{18}$O-enriched piperonol (3.4 mg, 22.0 µmol) in anhydrous dichloromethane (0.2 mL) under an argon atmosphere. 4Å molecular sieves powder (approx. 30 mg) was then added followed by stirring for 1 h. Tetra-*n*-propylammonium perruthenate (6.0 mg, 17 µmol) was added and allowed to stir for 1 h. An aliquot was then removed for MS analysis. The rest of the mixture was filtered through a plug of silica gel and flushed by 10% ethyl acetate in dichloromethane using argon. The suspension was then passed through a plug of silica gel and further flushed with 20% ethyl acetate in dichloromethane and then analysed by GCMS.

*Naturally abundant piperonal*

$^{1}$H NMR (500 MHz, $CDCl_3$) $\delta_H$ 9.80 (s, 1 H), 7.40 (dd, *J* = 7.9, 1.6 Hz, 1H), 7.32 (d, *J* = 1.6 Hz, 1H), 6.92 (d, *J* = 8.0 Hz, 1H), 6.07 (s, 2H); $^{13}$C NMR (100 MHz, $CDCl_3$) $\delta_C$ 190.2, 153.1, 148.7, 131.8, 128.6, 108.3, 106.9, 102.1; EIMS m/z (%): 63 (22), 65 (15), 91 (10), 119 (6), 121 (36), 149 (100), 150 (89), 151 (8); GCMS/MS piperonal $C_8H_6O_3$ calculated 150.0317; found 150.0311.

*$^{18}$O-enriched piperonal*

EIMS m/z (%): 61 (11), 62 (17), 63 (45), 64 (10), 65 (30), 91 (12), 93 (6), 121 (61), 149.1 (42), 150 (38), 151 (100), 152 (83) 153 (7); GCMS/MS $^{18}$O-piperonal $C_8H_6O_2{}^{18}O$ calculated 152.0359; found 152.0350.

*$RuO_22H_2O$*

A stock suspension of ruthenium dioxide was prepared by adding a stoichiometric amount of isopropanol to a concentrated solution (25.0 mM) of perruthenate in acetonitrile. Within minutes the dark green solution turned brown but the reaction was allowed to continue at 303 K for one hour to ensure quantitative formation of insoluble $RuO_2{\cdot}2H_2O$. Previous work has demonstrated that in organic solvent, secondary alcohols are oxidised by a stoichiometric quantity of perruthenate to give the ketone (in this case acetone) and $RuO_2{\cdot}2H_2O$.[4] An aliquot of the stock suspension was added to one of two pre-prepared cuvettes containing *n*-$Pr_4N[RuO_4]$ and NMO which were already thermostatted in the spectrometer at 303 K and the two reactions were initiated by a final addition

of diphenylmethanol. Acetone from the stock solution of the dioxide has no effect as it is an inert solvent which can itself be used for these oxidation reactions.[4]

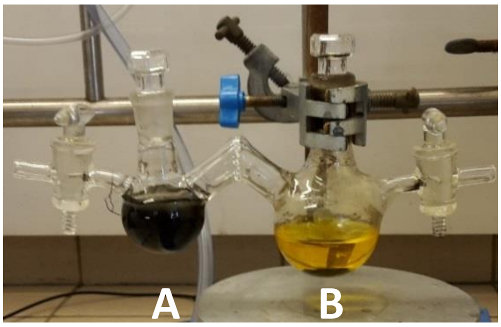


**Figure S1.** Experimental apparatus used to synthesise TPAP. Setup is shown post-reaction. Flask A contains TPAP product in solution.

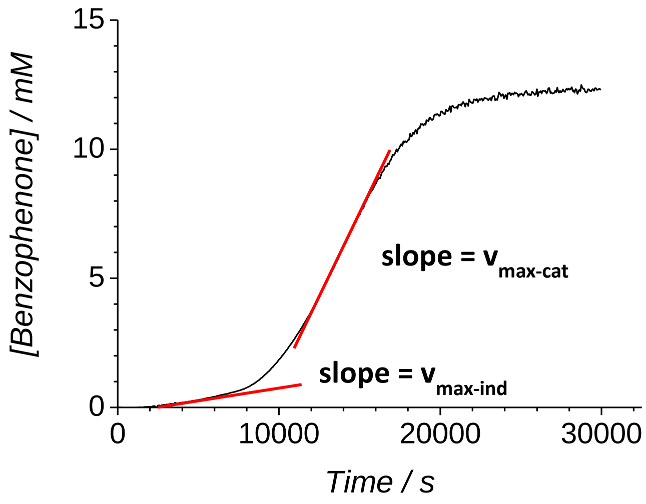


**Figure S2.** Maximum rate of oxidation during the induction and catalytic periods is determined by the slope of the steepest tangent within each region. Experimental conditions are identical to Figure 1 A.

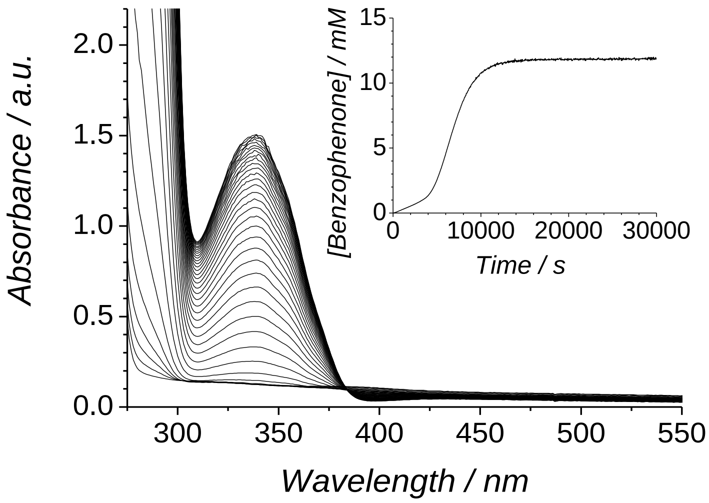


**Figure S3.** Time resolved spectra from Figure 1 with the constant spectrum of *n*-$Pr_4N[RuO_4]$ subtracted. Inset – benzophenone concentration *versus* time profile.

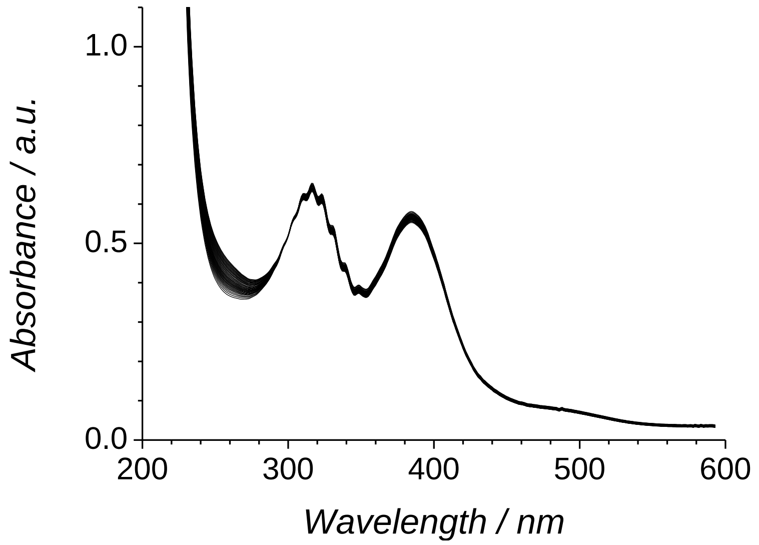


**Figure S4.** Time-resolved spectra following 0.25 mM *n*-$Pr_4N[RuO_4]$ in MeCN + 150 mM NMO over the course of seven hours (303 K). Spectra recorded every ten minutes. The spectrum is identical throughout to that of *n*-$Pr_4N[RuO_4]$ in MeCN.

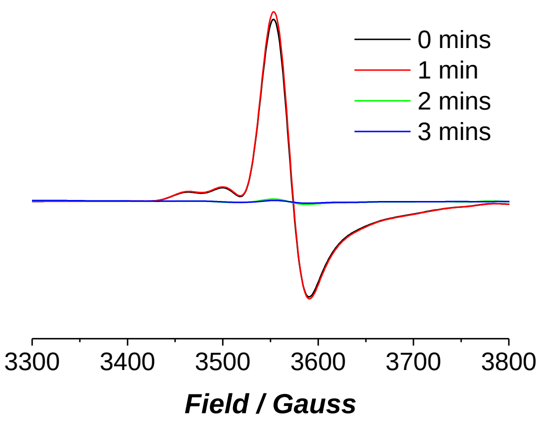


**Figure S5.** X-band ($\nu_{av}$ = 9.6766 GHz) CW EPR spectra measured at 6 K showing the decay of perruthenate EPR signal over time after addition of substrate alcohol in the absence of co-oxidant NMO.

### $^{18}$O Labelling of piperonol and its Ley-Griffith Oxidation

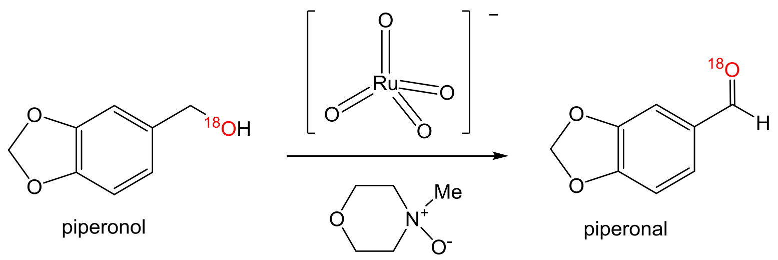


Mass spectral data Identified the starting material to be 78% $^{18}$O-enriched piperonol. After Ley-Griffith oxidation the MS data of product piperonal comprised a mixture of its molecular ion M and M-1 due to H-atom fragmentation at the aldehyde. Coupled with the mixture of $^{18}$O and $^{16}$O isotopomers derived from the 78% enriched starting material this leads to four possible compounds: $^{16}$O-piperonal-H (m/z 149), $^{16}$O-piperonal (m/z 150), $^{18}$O-piperonal-H (m/z 151) and $^{18}$O-piperonal (m/z 152) (Figure S7, S8). The $^{18}$O:$^{16}$O isotopic ratio was 83/38 (68% $^{18}$O enriched). Given the uncertainties in the mass spectral intensities, there is no significant change in the isotopic ratio going from piperonol to piperonal.

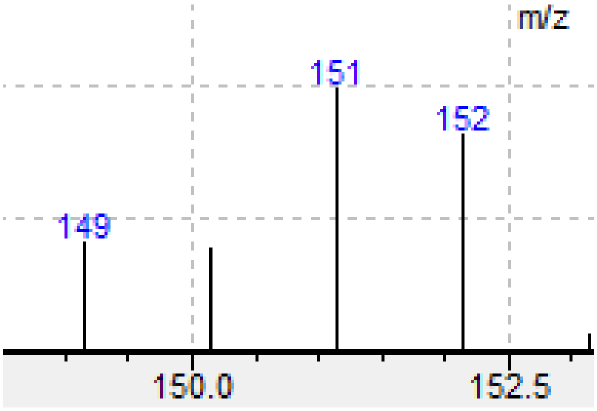


**Figure S6.** Mass spectra of the $^{18}$O-enriched piperonal product after Ley-Griffith oxidation. Various relevant structures on following page.

**1** 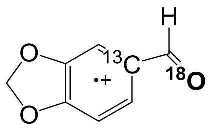 153

**2** 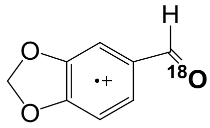 152

**3** 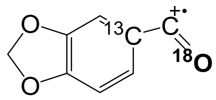 152

**4** 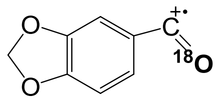 151

**5** 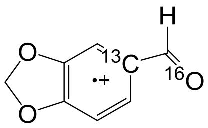 151

**6** 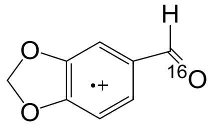 150

**7** 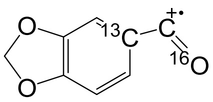 150

**8** 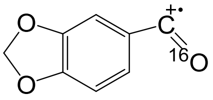 149

Mass fragmentation pattern with piperonol and piperonal.

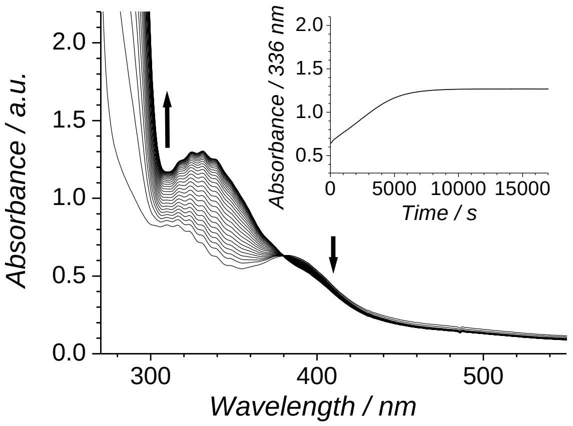


**Figure S7.** Time-resolved spectra following the oxidation of 6.0 mM diphenylmethanol by 0.25 mM *n*-$Pr_4N[RuO_4]$ (commercial, 97% pure) and 60 mM NMO in MeCN (303 K). Spectra are displayed at five minute intervals. Inset – Single wavelength profile at 336 nm.

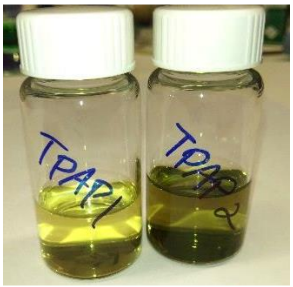


**Figure S8.** 1.8 mg of *n*-$Pr_4N[RuO_4]$ in MeCN (10 mL). Left – synthesised; Right – commercial.

**References**


1. D. D. Perin and L. F. Armarego, *Purification of Laboratory Chemicals*, 3rd edn., Pergamon Press, Oxford, England, 1988.
2. W. P. Griffith, S. V. Ley, G. P. Whitcombe and A. D. White, *J. Chem. Soc., Chem. Commun.*, 1987, 1625-1627.
3. W. Lin, L. long, D. Peng and C. Guo, *J. Organomet. Chem.*, 2007, **692**, 1619-1622.
4. A. C. Dengel, R. A. Hudson and W. P. Griffith, *Transition Met. Chem.*, 1985, **10**, 98-99.
